# The Effect of SnCl_2_/AmF Pretreatment on Short- and Long-Term Bond Strength to Eroded Dentin

**DOI:** 10.1155/2018/3895356

**Published:** 2018-04-19

**Authors:** Katrin Zumstein, Anne Peutzfeldt, Adrian Lussi, Simon Flury

**Affiliations:** Department of Preventive, Restorative, and Pediatric Dentistry, School of Dental Medicine, University of Bern, Freiburgstrasse 7, 3010 Bern, Switzerland

## Abstract

This study investigated the effect of SnCl_2_/AmF pretreatment on short- and long-term bond strength of resin composite to eroded dentin mediated by two self-etch, MDP-containing adhesive systems. 184 dentin specimens were produced from extracted human molars. Half the specimens (*n* = 92) were artificially eroded, and half were left untreated. For both substrates, half the specimens were pretreated with SnCl_2_/AmF, and half were left untreated. The specimens were treated with Clearfil SE Bond or Scotchbond Universal prior to application of resin composite. Microtensile bond strength (*μ*TBS) was measured after 24 h or 1 year. Failure mode was detected and EDX was performed. *μ*TBS results were statistically analyzed (*α* = 0.05). *μ*TBS was significantly influenced by the dentin substrate (eroded < noneroded dentin) and storage time (24 h > 1 year; *p* < 0.0001) but not by pretreatment with SnCl_2_/AmF or adhesive system. The predominant failure mode was adhesive failure at the dentin-adhesive interface. The content of Sn was generally below detection limit. Pretreatment with SnCl_2_/AmF did not influence short- and long-term bond strength to eroded dentin. Bond strength was reduced after storage for one year, was lower to eroded dentin than to noneroded dentin, and was similar for the two adhesive systems.

## 1. Introduction

A common method used to treat eroded teeth with exposed and sensitive dentin is to cover the eroded dentin with a sealant or an adhesive (with or without a resin composite) in order to prevent further loss of tooth substance [1–4]. It has been reported that the highest bond strength to eroded dentin is achieved by treating the dentin with a mild adhesive system (Clearfil SE Bond) following minimal roughening with a diamond bur [5]. However, eroded dentin is prone to adhesive failures. Compared to sound dentin, bond strength to eroded dentin is reduced [5, 6] and more adversely affected by aging [5].

One investigation found higher bond strength of said adhesive system Clearfil SE Bond to eroded dentin after treatment with a tin-chloride (SnCl_2_) solution than after treatment with a sodium-fluoride (NaF) solution or after no treatment [7]. The improvement in bond strength was tentatively explained by the fact that the organic matrix, though demineralized, retained Sn to a certain extent [8]. Sn ions were speculated to occupy negatively charged bonding sites such as phosphate groups in the organic matrix, thus making the collagen structure less polar and more accessible to the adhesive system. Clearfil SE Bond contains an acidic phosphate monomer (10-methacryloyloxydecyl dihydrogen phosphate (MDP)), which dissolves calcium from hydroxyapatite (HAP) and subsequently forms an MDP-calcium salt that may contribute to dentin bonding [9–11]. Sn ions, incorporated in collagen of the dentin, have been suggested to function as a calcium replacement and form an MDP-Sn salt and thus promote “docking” of MDP to collagen. A final mode of action was proposed: within the organic matrix and its collagen network, proteoglycans function as regulators for the three-dimensional arrangement of the collagen network and for the spacing of the collagen fibrils [12]. The removal of proteoglycans has been shown to enlarge spaces between collagen fibrils and to increase bond strength [13], and the authors speculated that these empty intercollagen spaces would be more accessible to an MDP-containing adhesive system such as Clearfil SE Bond. Proteoglycans can be removed by enzymes [13] but also by acids at low pH or relatively highly concentrated magnesium chloride (MgCl_2_; 0.5 M) [12]. Previous studies have shown enhanced adhesive performance when a SnCl_2_ solution (35%, pH < 1) was applied as an etching agent in combination with Clearfil SE Bond on sound enamel and dentin [14, 15]. Most recently, pretreatment with an amine fluoride (AmF) containing SnCl_2_ solution (SnCl_2_/AmF-solution; pH = 4.5) was found to improve the in vitro durability of Clearfil SE Bond coatings on sound, but smear-layer deprived dentin [16].

The aim of this study was to investigate the effect of SnCl_2_/AmF pretreatment on short- and long-term bond strength of resin composite to eroded dentin mediated by Clearfil SE Bond or by Scotchbond Universal, a more recently marketed one-step, MDP-containing adhesive. The null hypothesis was that there would be no difference in bond strength between the groups pretreated with SnCl_2_/AmF and the nonpretreated control groups regardless of adhesive system, artificial erosion, or storage time.

## 2. Materials and Methods

### 2.1. Preparation of Dentin Specimens

Sound, extracted permanent human molars were stored in 2% chloramine solution at 4°C until use. Before extraction, patients had been informed about the use of the molars for research purposes and verbal consent had been obtained. After extraction, the molars were pooled. The local ethics committee categorizes pooled teeth as an “irreversibly anonymized biobank” and, thus, no previous ethical approval was needed. The molars were cleaned under tap water and then embedded in circular molds with self-curing acrylic resin (Paladur, Heraeus Kulzer GmbH, Hanau, Germany) with only the root surfaces in contact with the self-curing acrylic resin. The molars were numbered consecutively and ground from the occlusal surface on a grinding machine with silicon carbide (SiC) paper grit #220 (Struers LaboPol-21, Struers, Ballerup, Denmark) until the entire surface was in mid-coronal dentin. The dentin surfaces were air-dried and carefully checked for absence of enamel, caries, and opening of the dental pulp. Subsequently, all dentin specimens were stored in the refrigerator at 4°C and 100% relative humidity.

A total of 184 extracted human molars were used, that is, 23 molars in each of eight groups that underwent one of eight pretreatment procedures: 20 molars for microtensile bond strength (*μ*TBS) measurements and three molars for EDX evaluation.

### 2.2. Artificial Erosion of Dentin Specimens

Half of the prepared dentin specimens underwent artificial erosion according to the following procedure: One hour before manipulation, the dentin specimens were retrieved from the refrigerator, stored in tap water, and then ground for 5 s on SiC paper grit #500 (Struers) to create a standardized smear layer. A new SiC paper was used after the grinding of ten dentin specimens. Between grinding and restoration the dentin specimens were constantly stored in tap water at room temperature. Subsequently, six demineralization-remineralization cycles per day were applied for 7 consecutive days, each cycle involving 5 min demineralization and 3.5 h remineralization. Between demineralization and remineralization the dentin specimens were rinsed with deionized water as previously explained [5]. The solutions for demineralization and remineralization are described in Table 1. The pH value of the solutions was checked daily. Following the artificial erosion procedure, the dentin specimens were stored in the refrigerator at 4°C and 100% relative humidity.

### 2.3. Pretreatment and Restoration of Dentin Specimens

One hour before pretreatment and restoration, the dentin specimens were retrieved from the refrigerator and stored in tap water at room temperature. Noneroded, sound dentin specimens were then ground for 5 s on SiC paper grit #500 (Struers) to create a standardized smear layer. A new SiC paper was used after the grinding of ten specimens. The eroded dentin specimens were not ground at this point so as not to damage the eroded surface. Each dentin specimen was rinsed with water spray and the peripheral enamel was removed with a diamond bur in order to ensure that the bonding surface was placed entirely in dentin. The dentin specimens were gently blow-dried. The eroded and noneroded dentin specimens then underwent individual pretreatment as described in Table 2.

After pretreatment, the dentin specimens were built up with two layers of 2 mm resin composite (Clearfil Majesty Esthetic, Kuraray, Okayama, Japan; color A4, Lot: 0008HA). Each 2-mm layer was light-cured for 30 s. For all steps of light-curing, an LED light-curing unit (Bluephase Polywave, Ivoclar Vivadent, Schaan, Liechtenstein) was used in “high power” mode, and the light power density was verified with a radiometer (Demetron LED Radiometer, Kerr Corporation) to be at least 1400 mW/cm^2^ at the beginning and end of specimen preparation. The restored dentin specimens were kept in 100% relative humidity and 37°C for 24 h (Memmert UM 500, Memmert & Co., Schwabach, Germany).

### 2.4. *μ*TBS Measurement and Failure Mode Determination

After the 24-h storage, the restored dentin specimens were sectioned with an electronically programmable diamond saw under water-cooling (Struers Accutom-5, Struers) perpendicularly to the adhesive interface in both *x* and *y* directions to obtain six beams from the most central part of each restored dentin specimen. Three beams per specimen were randomly selected for immediate measurement of *μ*TBS while the other three beams per specimen were kept at 100% relative humidity and 37°C for 1 yr (Memmert UM 500, Memmert & Co., Schwabach, Germany) before measurement of *μ*TBS. Prior to testing and in order to calculate the bonding surface (BSU (mm^2^)) of each beam, the width and breadth were measured using a digital caliper with an accuracy of 0.001 mm (IP 65, Mitutoyo, Kawasaki, Japan). The beams were then fixed by their ends to notched Ciucchi's jigs mounted in a universal testing machine (Syndicad TC-550, Syndicad Dental Research, Munich, Germany) with a low viscosity resin. The beams were stressed in tension at a crosshead speed of 1.0 mm/min until fracture and the maximum force (*F*_max_ (N)) was recorded. The *μ*TBS values (MPa) were calculated according to the formula *μ*TBS = *F*_max_/BSU.

The failure mode of each beam was stereomicroscopically determined at 45x magnification (Leica ZOOM 2000, Leica, Buffalo, NY, USA) and classified as (1) cohesive failure in dentin, (2) adhesive failure at the dentin-adhesive interface, (3) adhesive failure at the adhesive-resin composite interface, (4) mixed adhesive failure (failure modes 2 and 3), or (5) cohesive failure in resin composite.

### 2.5. Energy Dispersive X-Ray Spectroscopy (EDX)

For element analysis, 36 dentin specimens (*n* = 3 per group) were prepared as described above and underwent individual pretreatment as detailed in Table 2. After drying at ambient air, the specimens were analyzed unsputtered at 500-fold original magnification (JSM-6510, Jeol, Tokyo, Japan; acceleration voltage 15 kV). EDX-spectra were collected with a Silicon Drift Droplet Detector (X-Flash Detector 410-M; Bruker Nano GmbH, Berlin, Germany). The count rates were ~1 kcps and remained constant during the measurements. The elements carbon (C), oxygen (O), silicon (Si), phosphorus (P), and calcium (Ca) were quantified and relative values were given in % weight.

### 2.6. Statistical Analysis

The *μ*TBS results were analyzed statistically whereas failure mode and EDX results were evaluated descriptively. From the three *μ*TBS values obtained per dentin specimen for each storage time, a mean *μ*TBS value was calculated. Therefore, 20 *μ*TBS values per group (one mean *μ*TBS value per dentin specimen) were used for statistical analysis.

To test for significance of the factors “pretreatment,” “dentin substrate,” “storage time,” and “adhesive system” and for significance of their interactions, a global analysis of variance (ANOVA) was performed. A Shapiro Wilk's test (*p* = 0.6638) showed that the *μ*TBS values were normally distributed and, consequently, the *μ*TBS values were analyzed with a parametric ANOVA. The resulting *p* values were corrected for multiple testing with Bonferroni-Holm adjustment. No post hoc testing was needed because the ANOVA found no statistically significant interactions. The level of significance was set at 0.05.

## 3. Results

**Figure 1:**
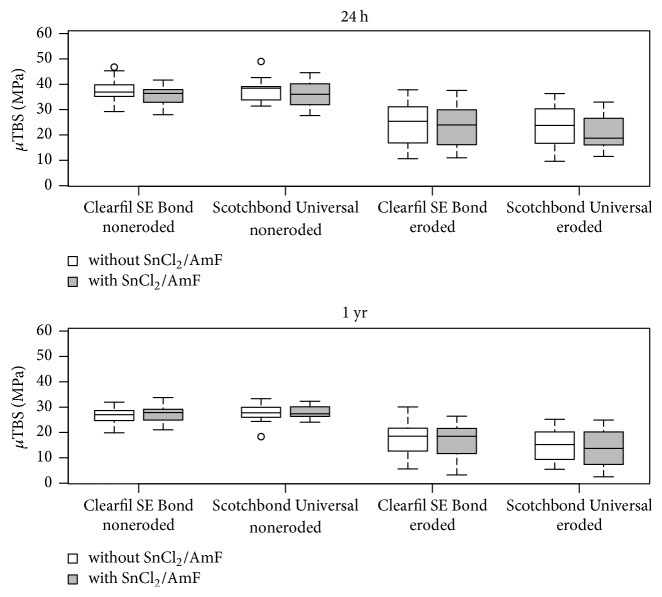
Microtensile bond strength (*μ*TBS; MPa; medians, lower and upper quartiles as well as minima and maxima) of Clearfil SE Bond and Scotchbond Universal to noneroded and eroded dentin after 24 h and 1 yr without or with SnCl_2_/AmF pretreatment (3 beams per dentin specimen and 20 specimens per group).

The *μ*TBS results are shown in Figure 1. There were a statistically significant effect of dentin substrate (*p* < 0.0001) and a statistically significant effect of storage time (*p* < 0.0001), but no statistically significant effect of pretreatment (*p* = 0.888) or adhesive system (*p* = 1.00).

Regarding the effect of dentin substrate, significantly lower *μ*TBS was found for dentin specimens that had undergone artificial erosion (*p* < 0.0001). The pooled means and standard deviations were 32.1 ± 5.9 MPa (noneroded dentin) and 19.6 ± 8.1 (eroded dentin). Regarding the effect of storage time, significantly lower *μ*TBS was found after storage for 1 yr as compared to after 24 h (*p* < 0.0001). The pooled means and standard deviations were 30.0 ± 9.2 MPa (24 h) and 21.7 ± 7.8 (1 yr).

The distribution of failure modes is shown in Table 3. In all groups, the predominant failure mode was adhesive failure at the dentin-adhesive interface. The second most common failure mode was mixed adhesive failure, but with two exceptions: the group of noneroded dentin pretreated with Clearfil SE Bond at both storage times presented more cohesive failures in resin composite, and the group of noneroded dentin pretreated with Scotchbond Universal and stored for 24 h presented more adhesive failures at the adhesive-resin composite interface.

**Figure 2:**
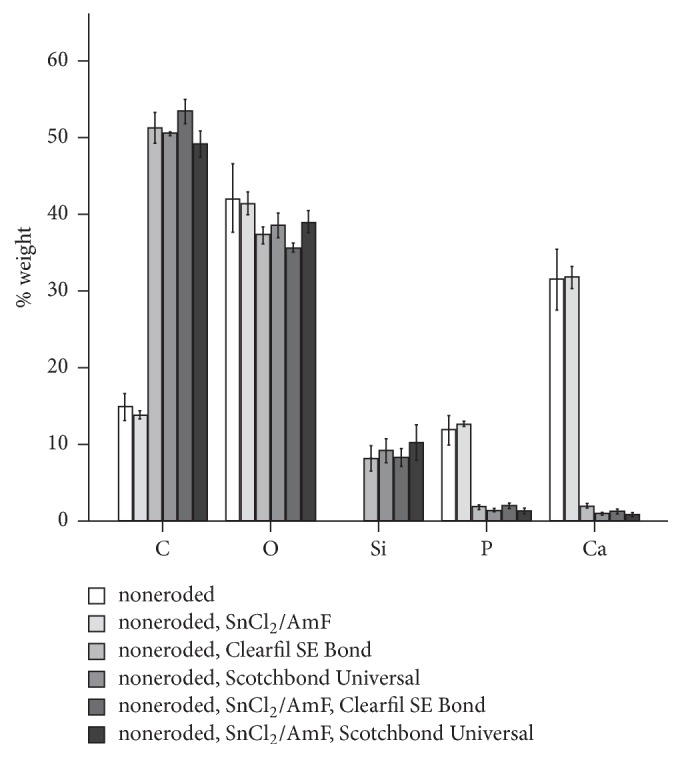
Percent weight of the elements carbon (C), oxygen (O), silicon (Si), phosphorus (P), and calcium (Ca) quantified with energy dispersive X-ray spectroscopy (EDX) for noneroded dentin specimens (*n* = 3 per group).

**Figure 3:**
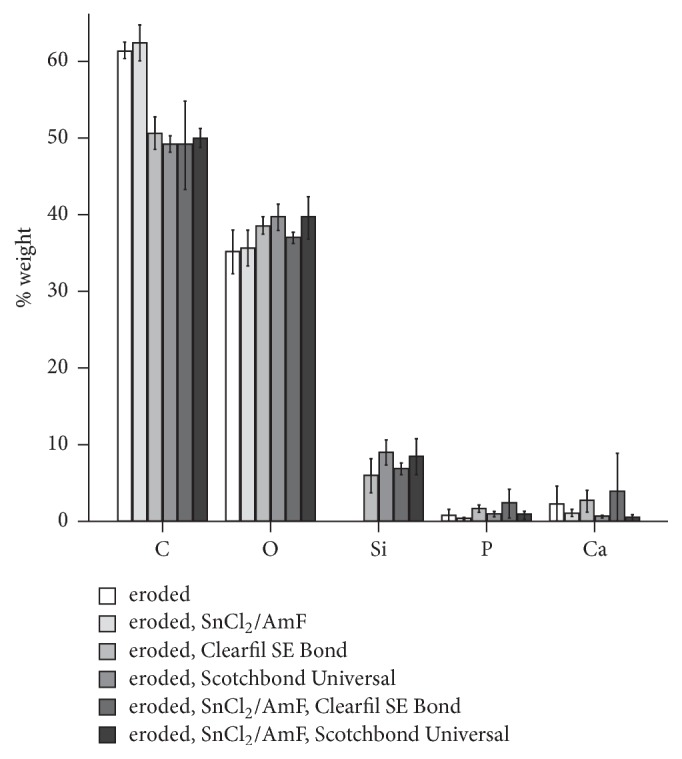
Percent weight of the elements carbon (C), oxygen (O), silicon (Si), phosphorus (P), and calcium (Ca) quantified with energy dispersive X-ray spectroscopy (EDX) for eroded dentin specimens (*n* = 3 per group).

The EDX results are displayed in Figures 2 and 3. Noneroded dentin, regardless of SnCl_2_/AmF pretreatment, presented the highest amounts of Ca and P and the lowest amounts of C. Treatment with Clearfil SE Bond or Scotchbond Universal increased the content of C in the surface, whereas only minor amounts of Ca and P were found. In addition, Si was detected. Eroded dentin, again regardless of SnCl_2_/AmF pretreatment, showed high amounts of C and low amounts of Ca and P. Treatment with Clearfil SE Bond or Scotchbond Universal led to a similar element distribution as in noneroded dentin. The content of Sn was generally below detection limit.

## 4. Discussion

The aim of this study was to investigate the effect of SnCl_2_/AmF pretreatment on short- and long-term bond strength of resin composite to eroded dentin mediated by Clearfil SE Bond or by Scotchbond Universal. First, bond strength was significantly lower to artificially eroded dentin than to noneroded dentin regardless of adhesive system and/or pretreatment with SnCl_2_/AmF. Therefore, the null hypothesis cannot be accepted as far as the factor artificial erosion is concerned. This finding is in accordance with those of previous studies [5, 6, 17]. Using TEM and the exact same artificial erosion protocol as in the present study, Zimmerli et al. [5] reported a thicker layer of exposed collagen that was only partially infiltrated by the adhesives applied. Likewise, Wang and Spencer [18] found the hybrid layer to be less complete, porous, and thicker than on noneroded, sound dentin. Collapse of the demineralized collagen fibrils and increased water content may not only have prevented the adhesives from fully infiltrating the demineralized zone [19] but may also have hampered their proper polymerization. Thus, the reduced bond strength to eroded dentin found in the present study was most probably caused by inferior hybridization. Considering the current results, it seems advisable to follow the recommendation made previously by Zimmerli et al. [5] to try and compensate for the reduced bond strength to eroded dentin by minimally roughening the eroded dentin with a diamond bur prior to application of the adhesive system [5]. Moreover, as shown by EDX, demineralization during erosion reduced the content of calcium and phosphorus in the eroded dentin specimens. Chemical bonding to dentin of the acidic phosphate monomer MDP is obtained mainly via calcium. Consequently, the reduced bond strength to eroded dentin compared to noneroded dentin and for both adhesive systems may have been caused not only by inferior hybridization of the eroded dentin, but also by the lower content of calcium.

Regarding the effect of storage time, significantly lower bond strengths were obtained following storage for 1 yr as compared to storage for 24 h. Therefore, the null hypothesis regarding the factor storage time cannot be accepted. This finding is in harmony with those of previous studies [20, 21]. Various factors have been held accountable for degradation of the resin-dentin interface such as the hydrophilic monomers incorporated in simplified adhesives, the water concentration in self-etch adhesives [21], inadequate infiltration of resin monomers within the hybrid layer, proteolytic breakdown of exposed collagen fibrils by activated endogenous collagenolytic enzymes [22], high permeability of the bonded interface, and phase separation within the hybrid layer [23]. Different agents such as matrix metalloproteinase inhibitors [24, 25] and collagen cross-linkers [26] are being tested for their ability to improve long-term dentin bonding.

The factor adhesive system had no significant effect on bond strength, and the null hypothesis was therefore accepted regarding this factor. The fact that the two adhesive systems proved equally capable of promoting a bond to dentin can be explained by the fact that both systems are self-etch adhesive systems containing MDP. It is then also not surprising that the two adhesive systems showed similar failure mode distribution, with a majority of adhesive failures at the dentin-adhesive interface. The results corroborate those of Amsler et al. [27] who also reported no significant difference between these two self-etch adhesive systems. Both adhesive systems are easy to apply. However, as Clearfil SE Bond is a “two-step” system, it requires an additional treatment step compared with the “one-step” system Scotchbond Universal and it thus imparts an additional source of error. On the other hand, Scotchbond Universal needs to be agitated for 20 s after application whereas the Primer of Clearfil SE Bond has to be left in place. This “agitation” step seems to be sensitive to handling as different operators may apply different force and different speed of agitation and it therefore constitutes a potential source of error.

Pretreatment of the dentin with SnCl_2_/AmF did not increase the bond strength, and the null hypothesis was therefore accepted regarding the factor pretreatment. The result is in contrast to those of previous studies [7, 14, 15], and the theory that Sn can be stored in completely demineralized organic matrix, that Sn can occupy negatively charged groups, and that Sn facilitates access for the adhesive system with help of a newly formed and less polar crystal lattice cannot be supported. One reason for the contrasting results could be that in the present study the organic matrix was not removed from the eroded dentin specimens; in one of the previous studies the demineralized layer in certain groups was removed with collagenase [7]. A co-explanation could be the storage time. Whereas Flury et al. [7] used a storage time of 24 h as in the present study, Peutzfeldt et al. [15] used a storage time of one week. Furthermore, it is possible that different concentrations and different pH-values of the SnCl_2_/AmF-solution had an influence on the results. Peutzfeldt et al. [15] used a 35% SnCl_2_/AmF-solution with pH < 1. Schlueter et al. [14] also used a concentration of 35%, but the pH was not indicated. Finally, Flury et al. [7] used an 800 ppm (0.08%) SnCl_2_/AmF-solution with a pH of 4.5 commercially available (elmex erosion protection dental rinse) and which was identical to the solution used in the present study except that the SnCl_2_/AmF-solution used in the present study was “home-made” and had no additives (e.g., flavoring agents, no chelating agents). The lack of effect on bond strength of pretreatment with the SnCl_2_/AmF-solution could in theory be explained by oxidation of the Sn^2+^ to Sn^4+^, which would have led to a loss of effect. As the EDX did not show any Sn^2+^ on the surface of the dentin specimens, Sn might have been inactivated before interacting with the dentin surface. However, further explorative *μ*TBS tests conducted using elmex erosion protection dental rinse and an identical study design also did not find any beneficial effect of the pretreatment on eroded dentin.

The analysis of failure modes showed that the predominant failure mode was adhesive failure at the dentin-adhesive interface which gives an indication of the weakest “link” in the adhesive interface. The fact that no cohesive failures in dentin or resin composite were observed indicates that the method applied (*μ*TBS) actually measured the strength of the adhesive bond and not the strength of the dentin or of the resin composite.

## 5. Conclusions

Based on the results of the present study, the following conclusions can be drawn:

- The two self-etch, MDP-containing adhesive systems Clearfil SE Bond and Scotchbond Universal led to similar bond strengths.
- The bond strength of resin composite promoted by the two adhesive systems was lower to eroded dentin than to noneroded dentin.
- The bond strength was reduced after storage for one year.
- Pretreatment with SnCl_2_/AmF had no influence on the bond strength.

## Figures and Tables

**Table 1:** Composition of demineralization and remineralization solutions (artificial erosion).

Solution	Composition
Demineralization	1% citric acid with pH 3.5 (anhydrous citric acid; Merck, Darmstadt, Germany)
Remineralization	0.002 g ascorbic acid, 0.58 g NaCl, 0.17 g CaCl_2_, 0.16 g NH_4_Cl, 1.27 g KCl, 0.16 g NaSCN, 0.33 g KH_2_PO_4_, 0.34 g Na_2_HPO_4_ dissolved in 1 l of demineralized water; pH set to 6.4 with HCl

**Table 2:** Pretreatment of dentin specimens.

	Clearfil SE Bond				Scotchbond Universal			
	Noneroded	Eroded	Noneroded	Eroded	Noneroded	Eroded	Noneroded	Eroded
			SnCl_2_/AmF pretreatment	SnCl_2_/AmF pretreatment			SnCl_2_/AmF pretreatment	SnCl_2_/AmF pretreatment
Step 1	Clearfil SE Primer^1^ 20 s	Clearfil SE Primer^1^ 20 s	SnCl_2_/AmF^4^ agitate 15 s	SnCl_2_/AmF^4^ agitate 15 s	Scotchbond Universal^3^ agitate 20 s	Scotchbond Universal^3^ agitate 20 s	SnCl_2_/AmF^4^ agitate 15 s	SnCl_2_/AmF^4^ agitate 15 s

Step 2	Gently blow dry 5 s	Gently blow dry 5 s	Water rinse 15 s	Water rinse 15 s	Gently blow dry 5 s	Gently blow dry 5 s	Water rinse 15 s	Water rinse 15 s

Step 3	Clearfil SE Bond^2^	Clearfil SE Bond^2^	Gently blow dry 5 s	Gently blow dry 5 s	Light-cure 10 s	Light-cure 10 s	Gently blow dry 5 s	Gently blow dry 5 s

Step 4	Gently blow dry 5 s	Gently blow dry 5 s	Clearfil SE Primer^1^ 20 s	Clearfil SE Primer^1^ 20 s			Scotchbond Universal^3^ agitate 20 s	Scotchbond Universal^3^ agitate 20 s

Step 5	Light-cure 10 s	Light-cure 10 s	Gently blow dry 5 s	Gently blow dry 5 s			Gently blow dry 5 s	Gently blow dry 5 s

Step 6			Clearfil SE Bond^2^	Clearfil SE Bond^2^			Light-cure 10 s	Light-cure 10 s

Step 7			Gently blow dry 5 s	Gently blow dry 5 s				

Step 8			Light-cure 10 s	Light-cure 10 s				

^1^Clearfil SE Primer, Lot: BJ0118, Kuraray, Okayama, Japan; ^2^Clearfil SE Bond, Lot: BC0191, Kuraray, Okayama, Japan; ^3^Scotchbond Universal, Lot: 586639, 3M ESPE, Neuss, Germany; ^4^SnCl_2_/AmF (800 ppm Sn^2+^, 500 ppm F^−^; pH = 4.5; RonaCare Olaflur, Merck, Darmstadt, Germany, Lot: L015039980).

**Table 3:** Distribution of failure modes (3 beams per dentin specimen; 20 dentin specimens per group).

Failure mode	Storage time	Clearfil SE Bond				Scotchbond Universal			
		Noneroded	Eroded	Noneroded	Eroded	Noneroded	Eroded	Noneroded	Eroded
				SnCl_2_/AmF pretreatment	SnCl_2_/AmF pretreatment			SnCl_2_/AmF pretreatment	SnCl_2_/AmF pretreatment
Cohesive failure in dentin (%)	24 h	0	0	0	0	0	0	0	0
	1 yr	0	0	0	0	0	0	0	0
Adhesive failure at dentin-adhesive interface (%)	24 h	38.3	46.6	61.6	83.3	33.3	65	66.6	85
	1 yr	51.6	76.6	66.6	93.3	61.6	88.3	60	93.3
Adhesive failure at adhesive-resin composite interface (%)	24 h	13.3	20	8.3	1.6	26.6	11.6	8.3	3.3
	1 yr	11.6	3.3	15	0	6.6	1.6	8.3	0
Mixed adhesive failure (%)	24 h	23.3	30	21.6	13.3	21.6	18.3	21.6	11.6
	1 yr	16.6	16.6	13.3	5	20	10	21.6	6.6
Cohesive failure in resin composite (%)	24 h	25	3.3	8.3	1.6	18.3	3.3	3.3	0
	1 yr	20	3.3	5	1.6	11.6	0	10	0
